# Personalized Medicine in Epidemics

**DOI:** 10.3390/jpm12040583

**Published:** 2022-04-05

**Authors:** Rutger A. Middelburg

**Affiliations:** Department of Public Health and Primary Care, Leiden University Medical Center, 2300 RC Leiden, The Netherlands; r.a.middelburg@lumc.nl

Before you lies the Special Issue “Personalized Medicine in Epidemics”. As we stated in our call for papers, we were looking for papers which make a novel contribution towards optimizing the personalization of medicine during epidemics. In this context, we aimed to include papers covering all kinds of epidemics, whether big or small, and whether infectious or non-infectious in nature. Personalized medicine is important in all fields of medicine, and all epidemics influence our ability to practice personalized medicine—not only for those suffering from the epidemic disease itself, but also for those with other diseases, which tend to get less attention as a major epidemic unfolds. This broadly inclusive view of the role of personalized medicine during epidemics has led to the inclusion of eleven high quality papers, on a wide range of topics within this Special Issue.

Dopazo et al. [1] review opportunities and challenges of personalized medicine in the context of the COVID-19 pandemic. Boboc et al. [2] were more specific in their approach, by reporting on their experience with diabetes mellitus type 1 in children during the COVID-19 pandemic. Besides an increased incidence, they also report important differences in patient characteristics between a pre-pandemic control group and the cases occurring during the pandemic.

Sticking with diabetes, Leutner et al. [3] report on different risk profiles and individual risk factors that predispose diabetic patients (predominantly type 2) to a whole range of diabetic complications. They further differentiated these associations between men and women. Meng et al. [4] report on the role of IgG N-glycan profiles in the prediction of progression from either isolated diabetes mellitus type 2 or isolated hypertension to a combination of diabetes mellitus type 2, hypertension, and diabetic comorbidity. They further differentiated these associations between different Chinese ethnic groups.

Bawadi et al. [5] investigated the relationship between different measures for abdominal fat and hypertension in adolescent males. Chang et al. [6] studied the association of serum urate concentrations with progression of kidney disease. They show a clear association for women, but not for men, suggesting different clinical strategies could be warranted for men and women.

Returning to infectious diseases, Tsai et al. [7] show that hepatitis C infection is associated with worse outcomes for diffuse large B-cell lymphoma, suggesting that direct-acting antiviral agents might help improve prognosis for this group of patients. Yang et al. [8] found that tuberculosis survivors who experience lasting ventilatory function disorders are more likely to also experience more respiratory symptoms, more limitations in physical activity, and a worse decline in quality of life.

Wu et al. [9] associated lung cancer prognosis with mode of detection (i.e., screen detected or non-screen detected), smoking status, and several other potential risk factors for poor outcome. For non-smokers, the screened status was one of the predictors, while it was not for smokers. Further, the probability of lung cancer being screen detected was much higher in non-smokers. Since smokers are also less likely to engage in screening, the authors suggest smokers’ prognosis might be improved by more effectively motivating them to participate in screening programs.

Finally, Hsiao et al. [10] identified several modifiable predictors of dental problems in children with disabilities, and Liu et al. [11] demonstrate that tongue pressure decline can be used as an indicator for chewing and swallowing problems in older adults.

Together, these eleven papers demonstrate the wide variety of epidemics in which personalization of medicine is affected. As personalization of medicine is important in all fields of medicine, so are all fields of medicine affected by epidemics. We therefore, after the successful completion of this first Special Issue on “Personalized Medicine in Epidemics”, now open our second Special Issue on this topic “Personalized Medicine in Epidemics 2.0” for submissions.
